# A Global Learning Agenda for the Levonorgestrel Intrauterine System (LNG IUS): Addressing Challenges and Opportunities to Increase Access

**DOI:** 10.9745/GHSP-D-18-00383

**Published:** 2018-12-27

**Authors:** Kate H. Rademacher, Tabitha Sripipatana, Anne Pfitzer, Anna Mackay, Sarah Thurston, Ashley Jackson, Elaine Menotti, Hayley Traeger

**Affiliations:** a FHI 360, Durham, NC, USA.; b United States Agency for International Development (USAID), Washington, DC, USA.; c Jhpiego, Baltimore, MD, USA.; d Marie Stopes International, New York, NY, USA.; e Population Services International, Washington, DC, USA.; f Seconded to WCG Cares by Population Services International, Seattle, WA, USA.; g USAID Global Health Fellows Program, Public Health Institute, Washington, DC, USA. Now with IBM Global Business Services, Washington, DC, USA.

## Abstract

The LNG IUS is one of the most effective forms of reversible contraception and has important noncontraceptive benefits but is currently not used at scale in any Family Planning 2020 focus country. A global working group developed a shared learning agenda to answer critical questions, harmonize approaches, avoid duplication, and facilitate introduction of the method within the context of informed choice.

## BACKGROUND

The levonorgestrel intrauterine system (LNG IUS) is one of the most effective forms of reversible contraception with efficacy rates similar to subdermal implants and copper intrauterine devices (IUDs).^1^ The LNG IUS is also associated with a number of important noncontraceptive health benefits, including treatment for menorrhagia (heavy menstrual bleeding) and uterine fibroids and potentially for anemia.^2^^–^^4^ In addition, as a result of the localized release of hormones and relatively low systemic blood levels compared with other hormonal methods, the side effects for the LNG IUS may be less pronounced than side effects with other hormonal contraceptives.^5^^,^^6^ (See Box 1 for a summary of the method's advantages.)

BOX 1 **Summary of Advantages of the LNG IUS**

- Highly effective contraceptive method
- Long-acting and reversible
- Rapid return to fertility after removal
- Can lead to reduced menstrual bleeding and cramping
- Localized release of hormones and relatively low systemic blood levels compared with other hormonal methods (side effects for the LNG IUS may be less pronounced)
- Removal can be easier than implant removals
- Noncontraceptive health benefits including treatment of heavy menstrual bleeding and potential reduction in iron-deficiency anemia
- May help protect against endometrial and cervical cancer
- No further action or supplies required once the LNG IUS is inserted
- Can be used immediately postpartum and post-abortion (Note: Manufacturers' labels for LNG IUS products do not currently include an indication for immediate postpartum insertions. However, the World Health Organization's *Medical Eligibility Criteria for Contraceptive Use* categorizes immediate postpartum insertion [<48 hours] of the LNG IUS as a category 1 [no restrictions] in non-breastfeeding women and as a category 2 [benefits outweigh the risks] in breastfeeding women.)

The LNG IUS has proved to be a popular choice with women in developed countries where the method is available, and it has helped revitalize the IUD market in some settings.^7^ Mirena, the 5-year LNG IUS product currently manufactured by Bayer Healthcare Pharmaceuticals Inc., was first introduced in the United States in 2000. At that time, less than 2% of women in the United States using contraception were using an IUD. Currently, almost 12% of contraceptive users have an IUD, and in 2014, 74% of women with an IUD were using a hormonal product.^8^ In 2015, the World Health Organization added the LNG IUS to its *Essential Medicines List*.^9^ Despite this and despite the method's advantages—which have been further described by colleagues (Hubacher^7^ and Jacobstein and Shelton^10^) previously in this journal— the method is not currently available at scale outside of the commercial sector in any of the Family Planning 2020 (FP2020) focus countries,^11^ and thus access to the larger population for this method is limited in these settings.

The LNG IUS is not currently available at scale in any of the FP2020 focus countries.

In this commentary, we review current challenges to LNG IUS access in low- and middle-income countries (LMICs). We then describe an introduction coordination platform that was launched in 2015 to help address these challenges and answer critical questions about the LNG IUS through a shared global learning agenda. We also discuss some of the advantages and disadvantages of this type of method-specific coordination platform and provide a call to action for other organizations that are considering introducing or scaling up the LNG IUS.

## BARRIERS TO ACCESS

### Product Costs

Historically, the high cost of LNG IUS commodities has been a primary barrier to public-sector procurement in international settings, and therefore to inclusion in the contraceptive method mix in national family planning programs.^7^^,^^10^ Mirena is offered on a very limited basis in private, for-profit settings in some developing countries. Recent market assessments conducted in Kenya, Madagascar, Nigeria, and Zambia have documented prices of Mirena to clients in urban settings ranging from US$60 to $400.^12^^–^^15^ In this price range, the method is prohibitively expensive for most women in LMIC markets.

An unbranded LNG IUS product manufactured by Bayer Healthcare is available for free by application through donations made by the International Contraceptive Access (ICA) Foundation, a private-public partnership between Bayer Healthcare and the Population Council. Since 2005, approximately 125,000 units have been donated through this mechanism in 36 countries.^16^ However, these donated units have generally been used to support small-scale pilot activities rather than to facilitate access through the health system on a regional or national scale. In addition, the ICA Foundation's LNG IUS product is registered in only a few countries, which means that a regulatory waiver must be secured when importing the product in most LMICs.

The landscape may be changing as new LNG IUS products become more available globally (Table). A new LNG IUS distributed by Medicines360, a nonprofit pharmaceutical company, was approved by the U.S. Food and Drug Administration in 2015,^17^ and the company is currently working with partners to register the product in FP2020 countries under the trade name Avibela. In early 2018, this product was approved in Madagascar and Zambia, and additional registrations are pending.^18^ The international public-sector procurement price for Avibela will vary by volume between US$12 to $16 per unit; for an order of 100,000 units, the public-sector price will be approximately US$15 per unit.^12^

**TABLE.** Overview of LNG IUS Products Approved by a Stringent Regulatory Authority^a^

Supplier	SRA-Approved LNG IUS Product	Availability and Pricing in FP2020 Countries
Bayer Healthcare	Mirena^b^	Mirena is provided commercially through private health care clinics in some developing countries on a very limited basis. Pricing between ∼US$60–$400 has been documented in recent market assessments in urban settings in Kenya, Madagascar, Nigeria, and Zambia.^12^^–^^15^
International Contraceptive Access (ICA) Foundation	Unbranded LNG IUS product	Through a public-private partnership between Bayer HealthCare and Population Council, a free unbranded LNG IUS product is provided by donation. Registered in several countries; brought in via regulatory waivers in other countries.
Medicines360	Sold in the United States under trade name Liletta; being registered in FP2020 countries under the trade name Avibela	The public-sector price to distributors for Avibela will vary by volume between US$12–$16; for an order of 100,000 units, public-sector transfer price will be approximately $15/unit. As of mid-2018, registered in Madagascar and Zambia.

Abbreviations: FP2020, Family Planning 2020; LNG IUS, levonorgestrel intrauterine system; SRA, stringent regulatory authority.

a In addition to the products listed in the table, there are several LNG IUS products that are being introduced in a limited number of FP2020 countries that are not currently approved by an SRA. As of 2018, there are no LNG IUS products that have been prequalified by the World Health Organization.

b Bayer Healthcare also manufactures the LNG IUS products Skyla and Kyleena. However, these products are not yet available in low- and middle-income countries, and therefore are not discussed here.

New, more affordable LNG IUS products are starting to become available in some FP2020 markets.

A recent assessment of the direct service delivery costs of various family planning methods per couple-years of protection (CYP) (inclusive of the cost for commodities, supplies, and provider time for insertion, resupply, and/or removal) demonstrated that at US$15 per unit, the cost per CYP of the LNG IUS compared favorably with that of other contraceptive methods commonly procured in FP2020 countries.^12^ This analysis used the assigned CYP factor of 3.3 years for an LNG IUS labeled for 5 years of use^19^; however, emerging evidence suggests that the duration of effectiveness of the LNG IUS is at least 7 years.^20^ In addition, Medicines360's clinical trial for the product is ongoing and will follow women for up to 10 years.^21^ If the duration of use is extended for the method, the CYP factor will increase and the cost per CYP will decrease even further.

Despite this, past experience with other methods demonstrates that even if a method is cost-effective over its duration of use, high upfront commodity costs can still be a barrier to procurement by donors and governments.^22^ Similar to the LNG IUS, contraceptive implants were not scaled up in LMICs for many years, partly because of high commodity costs. However, since the introduction of a more affordable 2-rod implant, Sino-implant (II)/Levoplant, and after the launch of the Implants Access Program—which was supported by a consortium of donors to lower the price of the 2-rod implant (Jadelle) and the 1-rod implant (Implanon/Nexplanon) and to support programmatic efforts to scale up access—implant use has grown rapidly in a number of FP2020 countries.^23^

The more affordable pricing for implants, which can be procured now by donors and governments for US$6.90 to $8.50 per unit in FP2020 countries, has set a new bar, which could impact expectations for pricing of the LNG IUS. In addition, copper IUDs are available for procurement for programs in FP2020 countries for less than US$0.50 per unit.^24^ (These prices do not reflect downstream costs such as import fees or shipping and distribution costs. Also, the actual cost to clients varies by service delivery outlet.) Interviews with key opinion leaders in several countries have indicated that if the upfront commodity cost of the LNG IUS remains substantially higher than that of other methods, particularly of implants, introduction and scale-up may remain challenging.^12^^–^^15^

### Demand and Service Delivery Considerations

As stakeholders consider whether to procure the LNG IUS for public-sector programs and/or invest in a global price reduction strategy, additional evidence is needed regarding the potential value of adding the LNG IUS to the method mix within countries. Stakeholders recognize that contraceptive commodity costs are only one factor impacting access and use. In the case of the LNG IUS, awareness and demand for the method would need to increase and potential supply-side barriers would need to be addressed for availability and uptake to expand substantially.^7^^,^^10^

Notably, while use of contraceptive implants has risen dramatically over recent years in sub-Saharan Africa, voluntary uptake of the copper IUD remains low with no country in the region having an IUD prevalence above 2%.^23^^,^^26^ A number of barriers limit voluntary uptake of the copper IUD, which may impact the potential of the LNG IUS as well. These factors include persistent myths and misperceptions about the IUD among potential clients and providers (for example, the incorrect belief that the method cannot be used by nulliparous women or that it causes infertility); the need for a pelvic exam; lack of provider competence and/or confidence in IUD insertion; a hesitancy among some providers to devote the extra time and effort required for IUD insertion relative to other contraceptive methods; lack of instruments and supplies; and low demand among women, which also reinforces supply barriers.^27^ Despite these challenges, there have also been some recent notable successes with copper IUD demand generation and provision, and use of the method has increased modestly in some countries in recent years.^23^^,^^28^^–^^30^ A key question about the LNG IUS is how the method would be positioned in relation to both the copper IUD and implants, given the similarities between the methods (e.g., all 3 require a certain level of provider training and skills; both the copper IUD and the LNG IUS require a pelvic exam and intrauterine insertion) and their differences (e.g., the LNG IUS is associated with reduced menstrual bleeding while the copper IUD is often associated with heavier menstrual bleeding).

The LNG IUS may face similar barriers to uptake as the copper IUD.

There is an emerging body of evidence about use of the LNG IUS in LMICs. For example, a recent study of LNG IUS users in Kenya found high satisfaction and continuation rates. Among 671 postpartum women offered a range of methods, 16% chose the LNG IUS.^31^ After 1 year of use, 89% of LNG IUS users were still using the method and 87% reported being very satisfied; these rates were comparable with those among users of implants.^32^ A separate qualitative assessment in Kenya documented experiences among early adopters of the Mirena and their male partners; a key finding was that women's main reason for choosing the LNG IUS was their perception that the method had fewer side effects compared with other contraceptive methods.^33^ A recent study in Nigeria documented perceptions of the method among clients, providers, and key opinion leaders in the country. In sites where the LNG IUS was introduced, the method represented less than 1% of all long-acting reversible contraceptives provided during the project time frame. Yet in qualitative interviews with LNG IUS users, providers, and key opinion leaders, the majority of respondents reported positive perceptions of the method. Findings from the study suggested that a comprehensive approach that addresses both demand- and supply-side factors will be required to gain traction with the method, and that while affordability of LNG IUS commodities is a prerequisite, lower prices alone will likely not be enough to translate into demand or uptake of the method at scale.^34^

Emerging evidence from several sub-Saharan African countries finds positive perceptions of the LNG IUS among users and providers.

Moving forward, additional evidence is needed to inform potential future introduction of the LNG IUS, especially to address the unresolved questions about demand. This will be particularly important given limited budgets and competing priorities as countries work to scale up the contraceptive methods they already have available. Specifically, questions remain about what demand generation and provider training strategies would be required to overcome potential barriers to uptake and whether the LNG IUS would be cost-effective compared with other long-acting reversible methods, especially if more resources are required to increase awareness and demand for the LNG IUS than are required for other methods. Governments and donors also want to know whether the method would primarily attract new users and/or “switchers.” Preliminary research in Kenya and Nigeria found that among switchers, a portion of LNG IUS users shifted from using short-acting resupply methods.^32^^,^^34^ If this outcome is replicated elsewhere, it will have important public health implications given that long-acting reversible methods like the LNG IUS have higher effectiveness and continuation rates than short-acting methods.^35^ In addition, more evidence is needed about women's perceptions and experiences of menstrual bleeding changes associated with the LNG IUS,^36^ as well as the potential impact of the method's noncontraceptive benefits including its potential to prevent or treat anemia.^7^

## DEVELOPMENT OF A GLOBAL LNG IUS LEARNING AGENDA

In 2015, a product introduction coordination platform, the LNG IUS Working Group, was convened by the United States Agency for International Development (USAID) with the goal of facilitating the introduction of high-quality, affordable LNG IUS products in developing countries in order to increase the range of highly effective contraceptive options available to women, within the context of informed choice. The LNG IUS coordination platform includes donors, suppliers, research agencies, and training and service delivery organizations that are currently supporting LNG IUS introduction and/or evaluation activities. The platform was formed to address the following objectives:

- Understand potential demand and use dynamics among different populations (such as new users and switchers), continuation rates, and client and provider perspectives of the method.
- Identify if and how the challenges that have impacted use of the copper IUD in many settings could be overcome by the LNG IUS, given the similarities and differences between the 2 methods.
- Identify effective strategies to generate awareness and demand for the LNG IUS including how to communicate method attributes in relation to attributes of other methods.
- Assess programmatic models in different service delivery channels.
- Evaluate willingness-to-pay for the LNG IUS among different market segments.
- Increase coordination among service delivery groups by sharing country introduction plans, service delivery approaches, and regulatory resources.
- Contribute to the global family planning community by making LNG IUS provider training materials, counseling materials, and job aids widely available.

As a first step, the group collaborated on development of a shared global learning agenda related to the LNG IUS. While the creation and use of a learning agenda is increasingly common in the development sector,^37^ we are not aware of any other formal efforts to collaborate with a diverse group of stakeholders to develop research and evaluation priorities for the LNG IUS in international settings. A primary goal of the process was to harmonize approaches and avoid duplication. Implementing partners in the group collaborated to develop a first draft of an LNG IUS learning agenda. Donors then further refined the list based on priorities and available resources. Next, the draft was shared with the larger LNG IUS Working Group, including LNG IUS manufacturers, for additional feedback and revision. The full learning agenda was approved and adopted by the LNG IUS Working Group in 2016 (Box 2). At the same time, members of the working group also recognize that the learning agenda is a “living document.” It is regularly revisited by the group and can be revised and updated as further evidence emerges from the field and/or when additional priorities are identified.

Members of the LNG IUS Working Group developed a shared global learning agenda for the LNG IUS.

BOX 2 **Global Learning Agenda for the LNG IUS**

**Learning Agenda Questions**

**A. Client Demand**

1. What are the profile(s) of the clients who will use this product?

a. Is there or would there be demand for this product among sub-populations with high unmet need for family planning (e.g., women in lower wealth quintiles, postpartum women, adolescents, post-abortion clients)?

b. Will introduction of the LNG IUS help reach new family planning users (i.e., current non-users)?

c. To what degree will introduction of the LNG IUS result in “switching” and from what other methods (e.g., from short-acting methods)?

2. Does the LNG IUS have the potential to ‘revitalize’ the IUD market in FP2020 countries?

a. Will demand for the LNG IUS be higher than demand for the copper IUD has been?

3. Would introduction of the LNG IUS increase family planning use overall/increase contraceptive prevalence rate(s)?

a. Can scale-up of this product help meet FP2020 goals?

4. How do continuation rates of the LNG IUS compare to continuation rates of other LARCs in multiple contexts?

5. Does immediate postpartum access to the LNG IUS increase use of postpartum family planning overall?

**B. Marketing**

6. What are effective demand creation strategies with different populations and in different sectors?

7. How can promotion of family planning including the LNG IUS be integrated into other health sectors such as nutrition programs or menstrual hygiene management programs?

**C. Service Delivery**

8. How can we overcome barriers that have impacted provision of the copper IUD at the service delivery level when introducing the LNG IUS?

9. What are health care providers' perceptions of this product?

10.What are effective service delivery models for LNG IUS provision? How does it differ by context, channel, and/or user group?

a. What are effective provider training strategies for the LNG IUS?

**D. Noncontraceptive Attributes**

11. How does knowledge of noncontraceptive attributes of the LNG IUS affect uptake and use?

a. What noncontraceptive attributes are most attractive to women in different contexts?

b. What noncontraceptive attributes are seen as most beneficial by providers in different contexts?

12. What are perceptions of amenorrhea among providers and various clients segments?

13. Can scale-up of the LNG IUS help reduce rates of anemia?

**E. Cost-Effectiveness and Pricing**

14. To what extent is the LNG IUS cost-effective compared to other family planning methods including other LARCs?

15. What is the willingness-to-pay for the LNG IUS among different populations of clients and different stakeholder groups?

Abbreviations: FP2020, Family Planning 2020; IUD, intrauterine device; LARCs, long-acting reversible contraceptives; LNG IUS, levonorgestrel-releasing intrauterine system.

## IMPLEMENTATION OF THE GLOBAL LNG IUS LEARNING AGENDA

Since its adoption in 2016, the LNG IUS learning agenda has been used in several ways:

- **Learning agenda questions were prioritized by donors and implementing agencies and used to inform investment and programming decisions.** The subcommittee of implementing agencies as well as donors involved with the LNG IUS Working Group each ranked the learning agenda questions in order of priority and relevance for their respective institutional strategies. This exercise was useful to identify how priorities differed among stakeholders and to facilitate further discussion among the group's members. The learning agenda also informed donor decisions for new research studies and maximized opportunities to leverage support among funders.
- **Research and service delivery groups coordinated to support data collection in pilot programs.** Members of the LNG IUS Working Group recognized that if all pilot programs introducing the LNG IUS could ask similar questions of women choosing the method as part of routine data collection, the impact of evaluation efforts could be increased. FHI 360 modified 3 questions that had been included in a research study in Kenya conducted by Hubacher and colleagues^31^^,^^32^ (Box 3). Service delivery groups including Jhpiego, Marie Stopes International, Population Services International, and WCG Cares then incorporated the questions into their pilot introduction efforts, with providers administering the questions in Kenya, Madagascar, Nigeria, Zambia, and Zimbabwe. Data were then compiled in a single dashboard so results could be compared and discussed. The dashboard will be updated on a regular basis as new data become available; the current version is available online through the Knowledge for Health platform.^38^ There were some challenges with implementation of this approach across countries and projects. For example, there were some discrepancies with how country programs implemented the questions (e.g., whether respondents were instructed to select a single answer to a question or whether multiple response options were possible). In addition, not all providers were willing or able to systematically collect the data from LNG IUS users given limited time and resources. Moving forward, it will be important to address these challenges and ensure that all groups more fully align on how the questions are asked. Despite the limitations to date, the approach has been successful in allowing programs to compare preliminary data about women's perspectives across various service delivery contexts.

BOX 3 **Coordinated Data Collection Approach Among LNG IUS Working Group Members in Multiple Countries**

Three questions were adapted from a previous research study in Kenya^31^^,^^32^ and were incorporated into programs where LNG IUS introduction activities funded by USAID were underway. In each project, a woman receives comprehensive counseling based on informed choice. If she chooses the LNG IUS, she is asked to consent to answer a version of the following 3 questions:

1. Can you briefly tell me the reasons you chose the LNG IUS today instead of another method?
2. If the LNG IUS had not been available today, what method, if any, would you have chosen instead?
3. How did you first find out about the LNG IUS?

Versions of these questions were administered by providers in Kenya, Madagascar, Nigeria, Zambia, and Zimbabwe. Data were then compiled by members of the LNG IUS Working Group in a single dashboard so results could be compared. The dashboard will be updated on an ongoing basis. The current version (as of November 2018) is available online in the IUD Toolkit on the Knowledge for Health platform.^38^

## ADVANTAGES AND DISADVANTAGES OF GLOBAL COORDINATION PLATFORM

The LNG IUS Working Group brings together partners that are supporting introduction and evaluation of the LNG IUS in pilot settings such that information shared during the meetings can be used immediately. In addition to providing a forum to define and implement the global learning agenda, the LNG IUS Working Group offers a platform to share updates and lessons from the field, training resources, and regulatory materials. The group has also facilitated approved sharing of LNG IUS product stock in country and provided an opportunity for members to co-develop client-centered counseling materials, such as a new job aid for providers about menstrual bleeding changes.^39^ In addition, the meetings serve as a platform for donors and manufacturers to obtain updates from multiple service delivery partners at the same time, allowing for an appreciation of the full scope of introduction activities and more immediate identification of common challenges that their input and resources can help resolve. The regular meetings also provide an opportunity to identify the evidence and funding gaps to shape existing project work plans and design future activities with an understanding of the key questions in the field that are not currently being investigated.

While the coordination platform has proved to be useful to participant organizations, it faces challenges that the group openly acknowledges. Suppliers with a product approved by a stringent regulatory authority are key partners in the LNG IUS Working Group, and they are not always able to share commercially sensitive information, especially among other suppliers. As such, some information and negotiation goes on in separate confidential meetings not open to the larger group. This limits the inclusion of all working group members in some planning activities; however, it ensures the continued involvement of manufacturers in this platform. The LNG IUS Working Group also includes service delivery and research partners that are potentially competing against each other for donor resources or a share of the LNG IUS market in countries. The donors involved have to be cognizant of the competitive nature of the work. For example, they must stay informed of when there are procurements being bid that may impact the working group participants' relationships while still holding partners accountable for coordination to ensure that LNG IUS introduction and evaluation efforts are as cost-effective and efficient as possible given limited resources.

## CONCLUSION

The implementation of a method-specific introduction coordination platform has allowed for the creation of a tailored learning agenda with input from diverse stakeholders. The LNG IUS is being introduced in contexts of informed choice, where it is one method among a range of contraceptives that country programs offer. There are learning gaps specific to the LNG IUS that have made coordination and collaboration useful for service delivery groups, research partners, manufacturers, and donors. Lessons from implementing this type of method-specific platform and global learning agenda model could be applied to other issues^40^ or other product introduction efforts.

At the same time, this type of single-method introduction coordination platform comes with costs as well as benefits. There are other new contraceptive methods being introduced in FP2020 countries, and the level of effort required for a single-method coordination group may not be needed or warranted in all cases. In addition, while the LNG IUS Working Group aims to apply experiences with scaling up access to other underused methods—such as implants and subcutaneous injectables—a platform that focuses on more than one method could further increase coordination and learning.

A single-method introduction coordination platform comes with costs as well as benefits.

Given the positive attributes of the LNG IUS and the potential benefits of adding it to the contraceptive method mix in LMICs, country-level stakeholders should consider if and when to introduce the method into family planning programs. At the same time, considering the potential programmatic challenges as well as unanswered questions such as the potential demand for the method if price barriers were removed, the new global learning agenda for the LNG IUS is a call to action for other entities engaged in LNG IUS introduction or research. We encourage other implementers and researchers to document and publish LNG IUS introduction experiences in LMICs, including uptake data, and to administer standardized monitoring and evaluation questions where possible. Rigorous research and program evaluations are essential, as are coordinated country-level introduction efforts, to better understand the potential impact of expanding access to this highly effective, potentially popular—yet now largely unavailable—contraceptive option.

## Notes

**Peer Reviewed**

**Funding:** This paper was made possible by the support of the American People through the United States Agency for International Development (USAID).
